# The Unc-5 Receptor Is Directly Regulated by Tinman in the Developing *Drosophila* Dorsal Vessel

**DOI:** 10.1371/journal.pone.0137688

**Published:** 2015-09-10

**Authors:** Jamshid Asadzadeh, Niamh Neligan, Judith J. Canabal-Alvear, Amanda C. Daly, Sunita Gupta Kramer, Juan-Pablo Labrador

**Affiliations:** 1 Smurfit Institute of Genetics, Trinity College Dublin, Dublin, Ireland; 2 Institute of Neuroscience, Trinity College Dublin, Dublin, Ireland; 3 Department of Pathology and Laboratory Medicine, Robert Wood Johnson Medical School, Rutgers, The State University of New Jersey, Piscataway, New Jersey, United States of America; National Institutes of Health (NIH), UNITED STATES

## Abstract

During early heart morphogenesis cardiac cells migrate in two bilateral opposing rows, meet at the dorsal midline and fuse to form a hollow tube known as the primary heart field in vertebrates or dorsal vessel (DV) in *Drosophila*. Guidance receptors are thought to mediate this evolutionarily conserved process. A core of transcription factors from the NK2, GATA and T-box families are also believed to orchestrate this process in both vertebrates and invertebrates. Nevertheless, whether they accomplish their function, at least in part, through direct or indirect transcriptional regulation of guidance receptors is currently unknown. In our work, we demonstrate how Tinman (Tin), the *Drosophila* homolog of the Nkx-2.5 transcription factor, regulates the Unc-5 receptor during DV tube morphogenesis. We use genetics, expression analysis with single cell mRNA resolution and enhancer-reporter assays in vitro or in vivo to demonstrate that Tin is required for Unc-5 receptor expression specifically in cardioblasts. We show that Tin can bind to evolutionary conserved sites within an Unc-5 DV enhancer and that these sites are required for Tin-dependent transactivation both in vitro and in vivo.

## Introduction

Early stages of heart development, both in vertebrates and invertebrates, include the migration of bilaterally paired condensations of cardiac precursors and the formation of a linear tube. The tube is formed once these symmetrical groups of mesodermal cells meet, and attach to each other leaving a luminal space between them [1, 2]. The coordinated migration of these mesodermal cells, bilateral interaction and the preservation of a lumen require complex interactions of multiple guidance receptors in *Drosophila* during DV morphogenesis [3–8]. Vertebrate homologs of the same ligand/receptor systems are expressed in the developing heart in many cases with strikingly similar patterns to the ones present in *Drosophila* [9, 10]. Some, like the Robos and their Slit ligands [11, 12] or plexins and semaphorins [13, 14], have also been identified as key players at different stages of heart development. Nevertheless, how these guidance systems are regulated in place and time during heart morphogenesis is widely unknown.

Cardiogenesis in both vertebrates and invertebrates also requires the key regulatory actions of a core of evolutionarily conserved families of transcription factors (NK2, GATA, and T-box) [15]. They are required early in development during linear tube formation and function again at later stages of heart morphogenesis in vertebrates [16]. For example, Nkx2-5 members and its *Drosophila* homolog, Tinman (Tin), play an important role in early cardiogenesis starting with the specification of cardiac precursors to remodeling and functionality of the adult heart [1, 17]. Given the role of guidance systems in heart morphogenesis, it is likely that they are direct or indirect targets of these families of transcriptional regulators.

**Fig 1:**
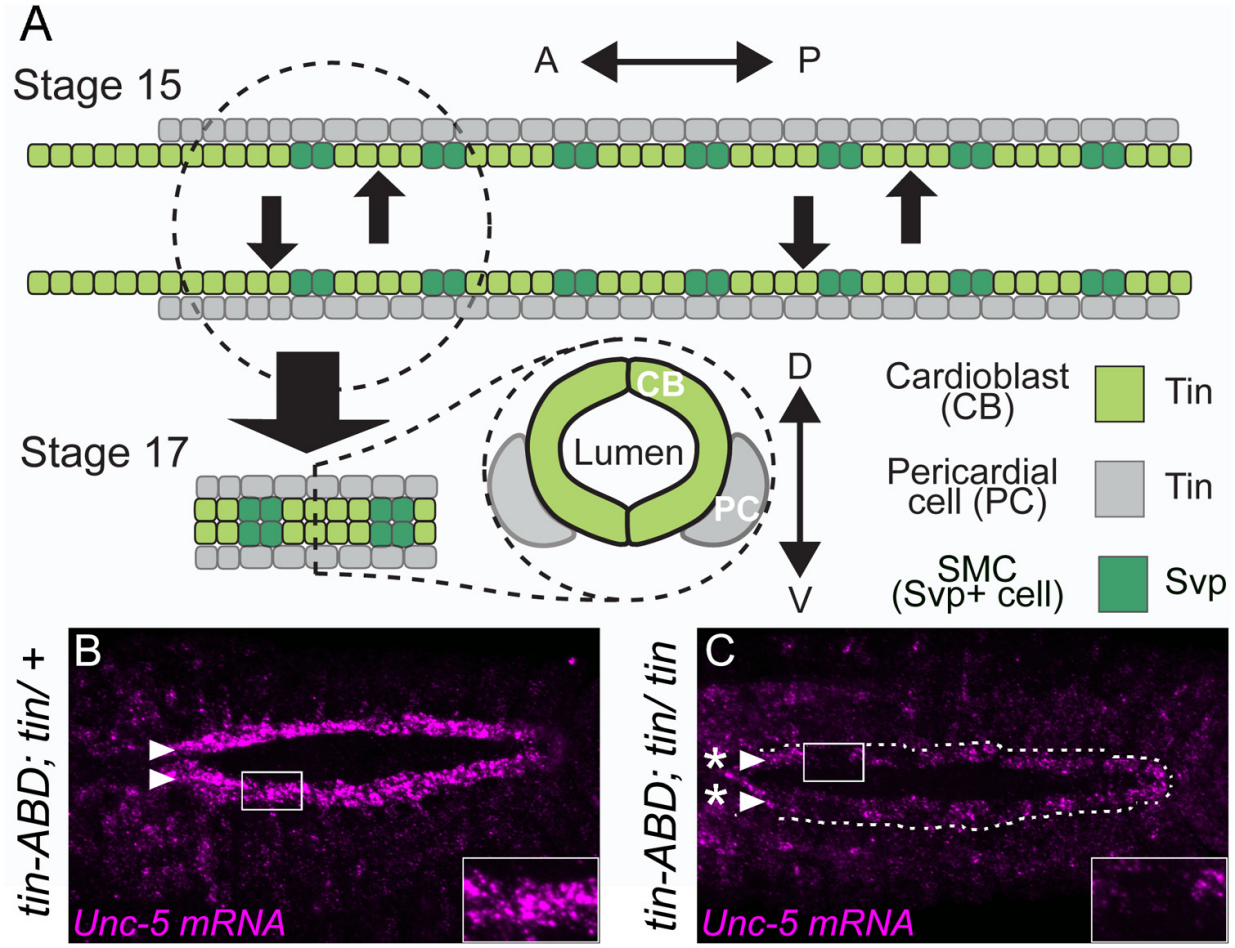
*tin* regulates *Unc-5* expression in vivo. (A) Organization and cellular composition and development of the Drosophila dorsal vessel. Schematic represents Drosophila DV at embryonic stages 15 (migrating cells, top) and 17 (tubular heart already formed, bottom) with different cell types color-coded based on the marker TF expressed. Aortic portion is oriented anteriorly and the beating portion (heart) posteriorly. CBs are divided into Tin- (light green) or Svp-expressing SMCs (dark green) subtypes. The three most posterior pairs of Svp-expressing CBs will make the future ostial (inflow valve) cells. Pericardial cells (gray) surround the CBs on their ventrolateral side and fall into two major types; Tin-positive or Tin-negative PCs (not colored). Bottom is a schematic cross section of the heart lumen at stage 17 where CBs on opposite sides take a crescent-like shape after contact, leaving in between them a hollow luminal space. (B) *Unc-5* mRNA (magenta) is present in the DV in embryos heterozygous for *tin* ^*346*^ (B). In *tinABD;tin* ^*346*^ */tin* ^*346*^ homozygous mutant embryos, however, *Unc-5* mRNA expression is significantly reduced (C). Anterior side of the embryo is to the left in panels B and C.

To gain a better understanding if these transcription factors (TFs) control heart tubulogenesis through the regulation of guidance receptors, we have studied Unc-5 receptor’s regulation in the *Drosophila* dorsal vessel (DV). The DV develops from mesodermal cardiac precursors. After precursor division, heart cells line up bilaterally into two rows where myocardial cells or cardioblasts (CBs) are positioned dorsally and pericardial cells (PCs) ventrolaterally. Finally, they migrate together towards the dorsal midline of the embryo where CBs fuse to form the tubular heart (Fig 1A). The CBs will constitute the pumping myocardium and PCs the pericardium. The *Drosophila* Unc-5 receptor is a repulsive receptor for Netrin A and B [18, 19] and in the nervous system has been shown to be required for motoneuron guidance [20, 21] and glial migration [22]. Unc-5 is expressed in both major cell types present in the DV and has recently been shown to be required in late dorsal vessel morphogenesis for lumen formation [7, 8]. *tin* is also expressed widely in PCs and most CBs (Fig 1A). Furthermore, *tin* and *Doc*, a T-box family TF, have been shown to regulate together an early cardiac mesoderm *Unc-5* enhancer [23]. In this work we show that *tin* is specifically required for *Unc-5* expression in CBs and is sufficient to induce its expression ectopically in the ectoderm. We identify a unique DV enhancer within the *Unc-5* regulatory region that fully recapitulates its expression at late stages of DV fusion. Cardioblast specific expression through this enhancer is strictly dependent on *tin* as is misexpression in the ectoderm. Additionally, Tin can induce transcription in vitro in a luciferase assay through the *Unc-5* DV enhancer but not from other known *Unc-5* enhancers. Using ChIP analysis we identify three evolutionary conserved Tin-binding sites within this enhancer that are required in vitro for its activity. Finally, we demonstrate that these sites are the Tin-binding sites required in the *Unc-5* DV enhancer for its ectopic regulation in the ectoderm and, more importantly, its specific expression in cardioblasts. Thus, Our work shows how *tin* regulates Unc-5 receptor expression during late heart tube morphogenesis when Unc-5 is required for lumen formation. Our results provide a regulatory mechanism for a guidance receptor through a direct interaction with three conserved sites within its DV enhancer by one of the core transcription factors during tubulogenesis of the Drosophila DV.

## Results

### Tin is required for Unc-5 expression in the dorsal vessel

The Unc-5 receptor localizes preferentially at the luminal side in CBs at the onset of tubulogenesis and it is required to preserve the luminal space between CBs [7, 8]. However, how this receptor is regulated at this late stage is not known. Genome-wide chromatin immunoprecipitation screens to identify Tin target genes in cardiac mesoderm and cardiac precursors have identified *Unc-5* as one of its targets [23, 24]. Previous studies have established that early cardiac specification in *Drosophila* is dependent on the homeobox transcription factor Tin [2, 25, 26]. As a consequence, *tin* loss-of-function mutants lack a DV due to its early role in the mesoderm to specify cardiac progenitors [26, 27]. Nevertheless, in cardiac specific *tin* mutant animals (*tin-ABD*;*tin* ^*346*^/*tin* ^*346*^), where *tin* is re-expressed in a mutant background under the control of enhancer elements (ABD) recapitulating its entire endogenous expression pattern except in the dorsal vessel. Myocardial cells are specified and the DV forms in these mutants [17]. To determine whether *tin* regulates *Unc-5*, we analyzed its expression in *tin* mutant DVs (Fig 1B and 1C). *Unc-5* mRNAs is significantly reduced when compared with *tin* heterozygous DVs (compare 1B with 1C). Thus, *tin* is required for *Unc-5* expression in the DV at the onset of tubulogenesis.

### Tinman is sufficient to induce Unc-5 expression ectopically

**Fig 2:**
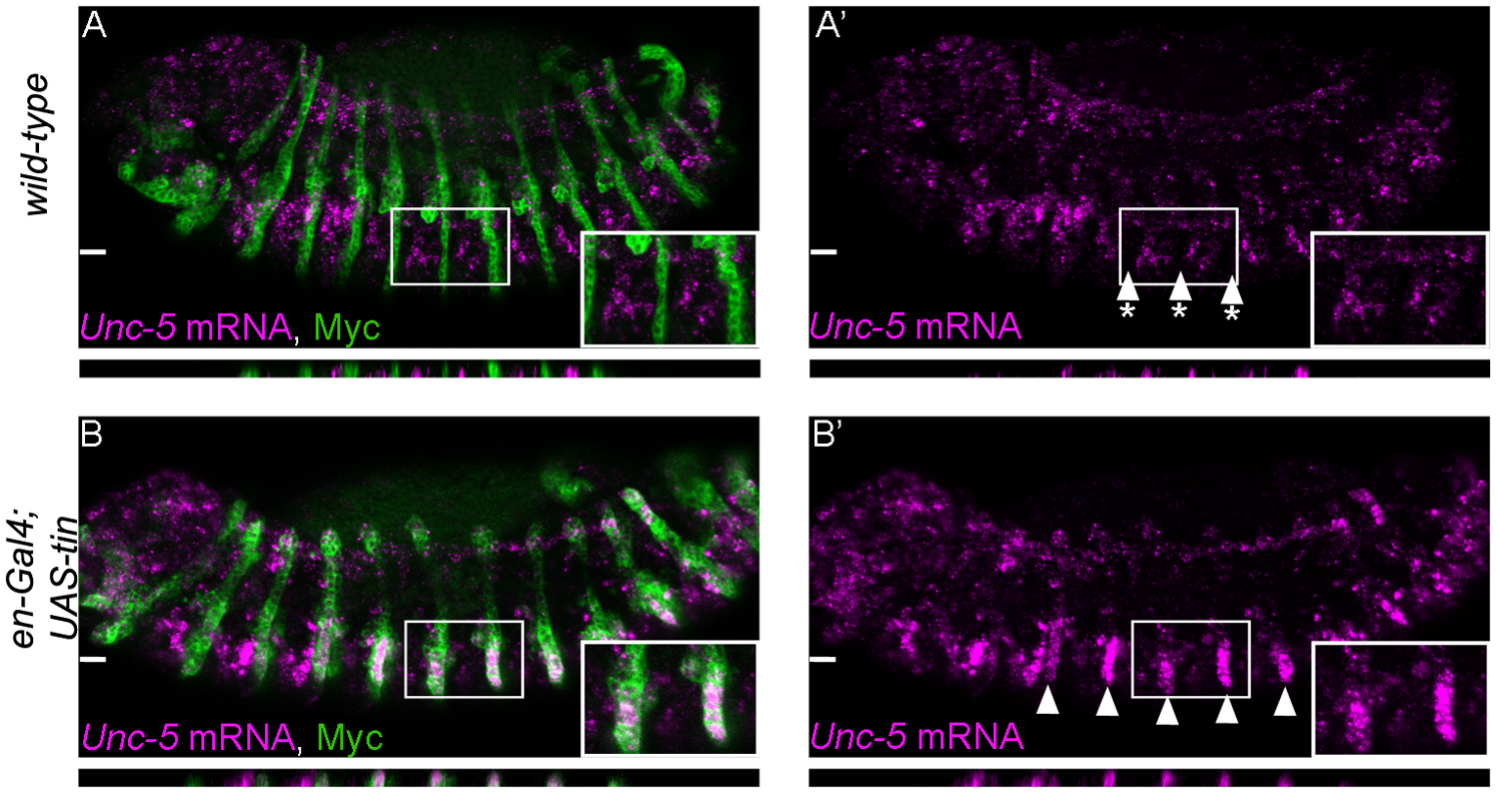
Ectopic expression of Tin induces *Unc-5* mRNA expression in vivo. (A-A’) in situ hybridization for endogenous mRNA expression of *Unc-5* does not show any striped ectodermal signal (magenta, arrowheads with asterisks). (B–B’) ectopic expression of *tin* in ectodermal stripes (green) from a *UAS-tin* transgene using *engrailed-Gal4* induces endogenous *Unc-5* mRNA in a striped pattern (compare A’ with B’, arrowheads). Engrailed stripes are labelled green by co-expression of Tau-Myc from a *UAS-tau-myc* construct. All panels are lateral views with anterior to the left. A magnification of the regions delineated by insets is shown for each panel. *Unc-5* in situ fuorescence in *engrailed* stripes was quantified (0.33±0.06 s.e.m. or -0.03±0.01 s.e.m. in *tin*-expressing *en*-stripes A, A’ or *en*-stripes not expressing *tin* respectively B, B’, p<0.005, n = 12). Confocal XZ sections are presented below each panel (location of the sections is indicated in the upper panel a white line) where Unc-5 specific signal can be detected colocalizing with *en*-stripes when tin is expressed (B, B’) but not in a *wild-type* embryo (A, A’).

We further tested *tin*’s sufficiency to induce *Unc-5* transcription *in vivo* by misexpressing *tin* in ectodermal stripes with an *engrailed-Gal4* (*en-Gal4*) driver. We detected *Unc-5* mRNA through in situ hybridization and we confirmed that, indeed, ectopic *tin* expression in the ectoderm is sufficient to induce *Unc-5* in the characteristic *engrailed* stripes (Fig 2B and 2B’) where neither *tin* nor *Unc-5* are normally not expressed (Fig 2A and 2A’). Thus, *tin* is not only required for *Unc-5* expression in the dorsal vessel but it is also sufficient to induce its expression in other tissues.

### Identification of the Unc-5 cardiac enhancer

**Fig 3:**
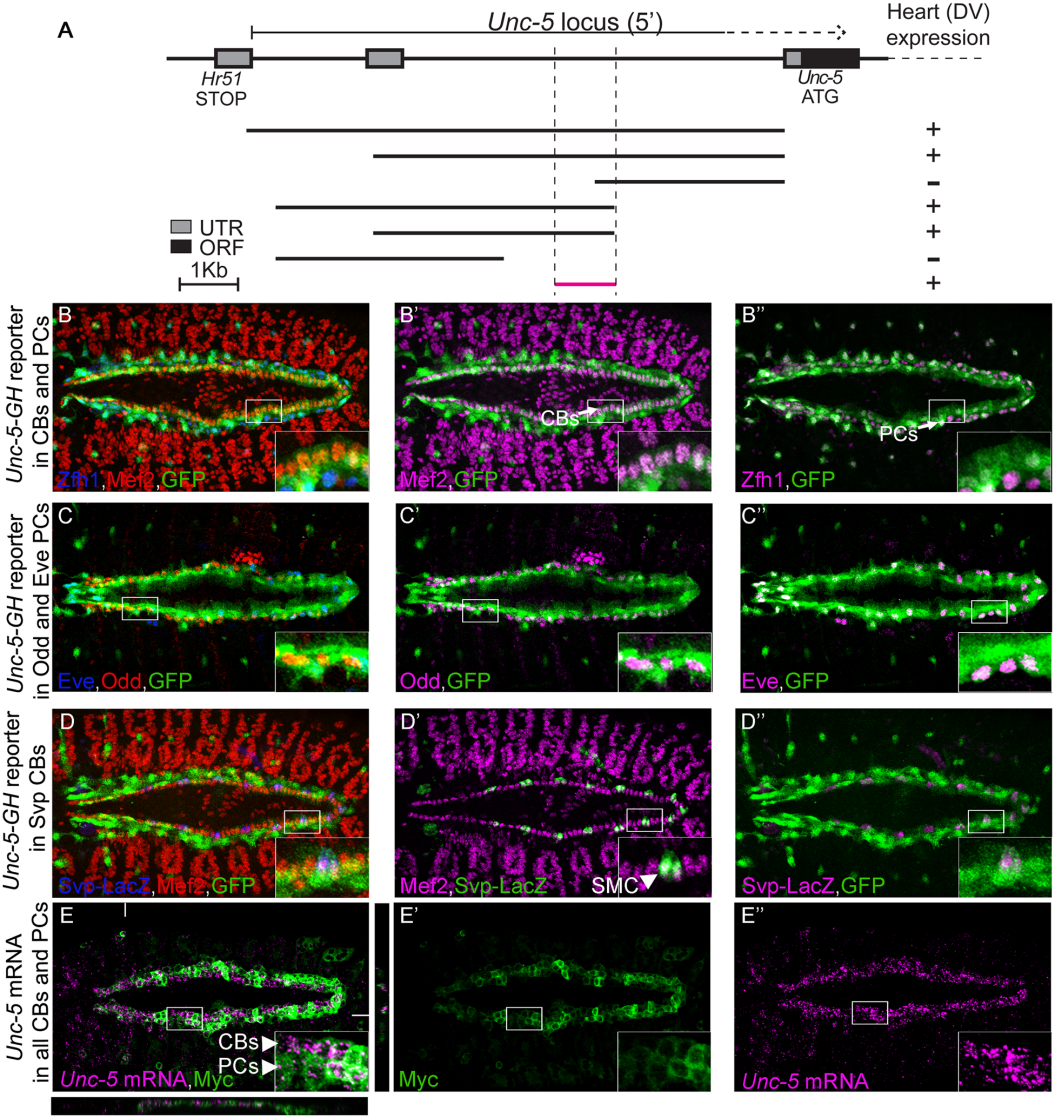
The *Unc-5-GH* reporter is expressed in all cardioblasts and pericardial cells. (A) Schematic representation of the positions and the relative sizes of the dissected fragments from the *Unc-5* locus. Reporter constructs were generated by fusing each fragment to a GFP open reading frame. Examination of GFP expression in transgenic lines carrying any of these reporters indicates potential enhancer activity of each fragment. All reporters containing the smallest (bottom) fragment revealed activity in the DV. The smallest fragment (GH-reporter) was chosen for further examinations. (B-D”) We used specific markers to label CBs: Mef2, (B and B’, [29]), PCs: all PCs with Zfh1 (B and B”,[30, 31]) or subsets with Eve and Odd (C-C”, [32, 33]); and a Svp-LacZ reporter for labelling a set of Tin-negative myocardial cells, also known as Seven Up (Svp)-positive myocardial cells (SMCs) (D-D”, [17, 34]). GFP expression (green) is present in all CBs (B’; magenta) and PCs (B”; magenta). (C–C”) Odd- (C’; magenta) and Eve-positive (C”; magenta) PCs express GFP (green) driven by *Unc-5-GH* enhancer fragment. (D–D”) *Unc-5-GH* reporter also drives expression in Tin-positive CBs and Svp myocardial cells (SMC). (E-E”) Correlation between GH enhancer expression pattern and endogenous *Unc-5* mRNA expression in the DV was examined by in situ hybridization. Colocalization of Tau-Myc expression pattern (labeled in green), driven by the enhancer (*Unc-5 GH-Gal4*), and *Unc-5* mRNA (magenta) indicates perfect overlap between the two. XZ an YZ sections are presented at the bottom and right of the main panel (E) and white lines indicate their location. CB cardioblast, PC pericardial cell. All panels are dorsal views with anterior to the left. A magnification of the regions delineated by insets is shown for each panel. All embryos are stage 15.

In order to identify regulatory regions required for *Unc-5* expression in the DV, we dissected the *D*. *melanogaster Unc-5* genomic locus into overlapping fragments of varying length starting from the preceding gene (*Hr51*) to the 5th intron within the *Unc-5* locus [28] (Fig 3A). All the fragments were fused to a GFP ORF and inserted into the same locus to avoid any variability due to position effect. We identified a unique 1kb minimal fragment upstream of the *Unc-5* ATG sufficient to drive GFP expression in the DV at late stages of PCs and CBs migration and during tube formation (stage 13 onwards, Fig 3A) largely overlapping with the early mesoderm enhancer previously described [23]. To characterize the expression pattern of this enhancer we co-stained embryos carrying the DV enhancer driving GFP (GH reporter) with anti-GFP and markers for PCs or CBs, Mef2. The reporter drives GFP expression in all Tin-expressing CBs and PCs (Fig 3B–3D”) including Eve- and Odd-positive PCs (Fig 3C–3C”) and the Tin-negative Seven Up (Svp)-expressing myocardial cells (SMCs, Fig 3D–3D”). Finally, to confirm that the enhancer faithfully recapitulates *Unc-5* endogenous expression in the *Drosophila* DV we also performed double labeling of the cells expressing the reporter and *Unc-5* mRNA by in situ hybridization. Our data shows that the *Unc-5* mRNA expression pattern in the DV fully matches that of the enhancer (Fig 3E–3E”).

### *tin* is required for activation of the *Unc-5* DV enhancer in cardioblasts

**Fig 4:**
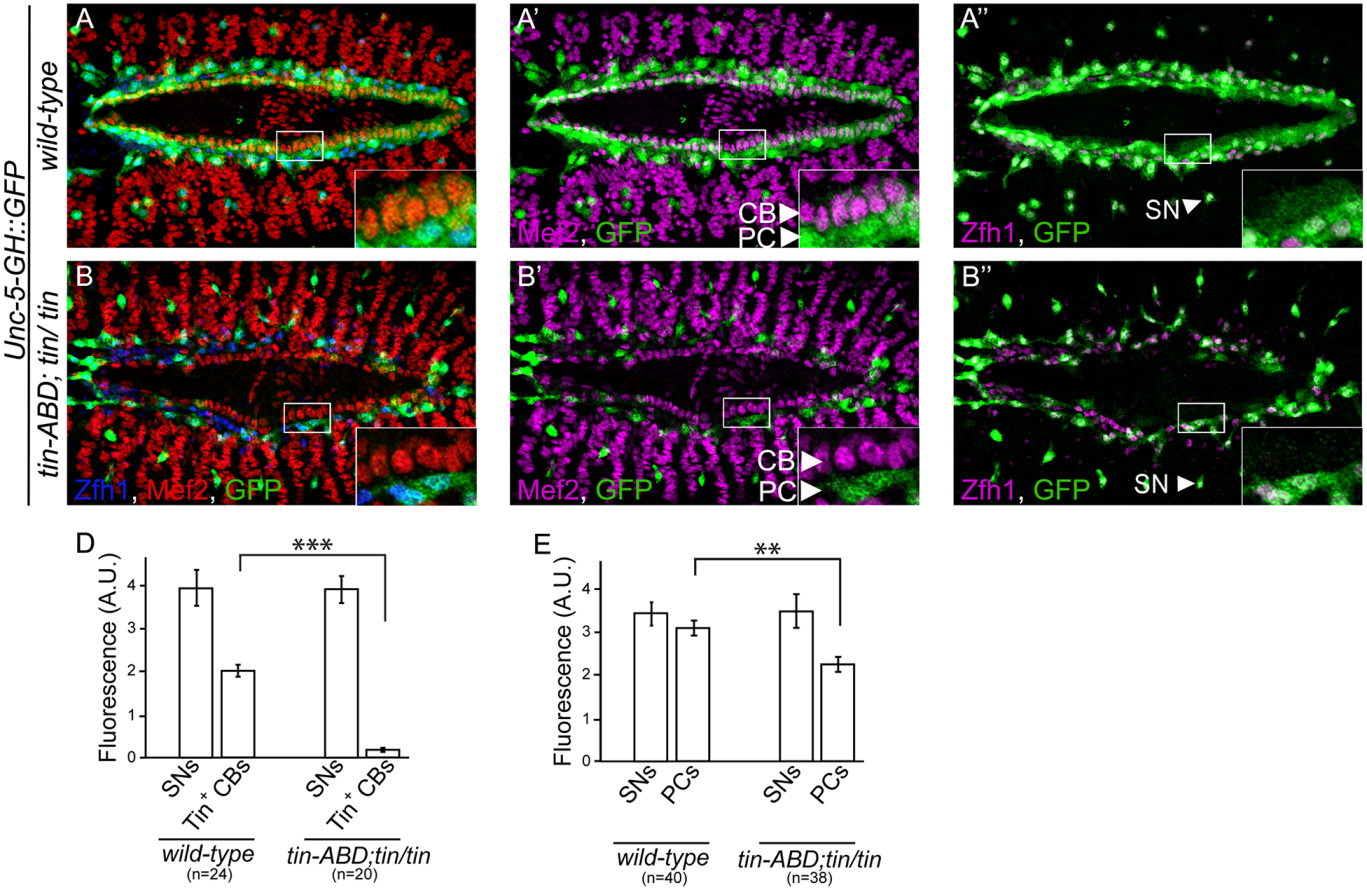
The *Unc-5* heart enhancer element is regulated by *tin in vivo*. (A–A”) Reporter gene (green) is expressed in all CBs and PCs in *wild-type* embryos. (B–B”) In *tin-ABD*; *tin* ^*346*^/*tin* ^*346*^ mutant embryos, where *tin* is only absent in the DV, reporter gene expression is almost absent in CBs while it is only partially downregulated in PCs. Note the unchanged GFP expression in sensory neurons (arrowheads in A” and B”) in *wild-type* and mutant backgrounds. Quantification of GFP expression in CBs (D) or PCs (E). Genotypes of embryos are indicated on the X axis and fluorescence on the Y axis. GFP expression in SNs is used as internal control and their fluorescence is not affected in *tin-ABD*; *tin* ^*346*^/*tin* ^*346*^ mutant background (3.86 ± 0.4 s.e.m and 3.82 ± 0.29 s.e.m, in *wild-type* or *tin* mutants respectively). However, the signal is significantly (*P*<6 x 10^−14^) reduced in CBs, from 1.98 ± 0.128 to 0.173 ± 0.04 (D). PCs show a slight reduction in signal (E), from 3.4 ± 0.2 in *wild-type* to 2.47 ± 0.21 in *tin-ABD;tin* ^*346*^ (*P*<6.2 x 10^−4^). All panels are dorsal views with anterior to the left. A magnification of the regions delineated by insets is shown for each panel. All embryos are aged from early to late stage 15. CB cardioblast, PC pericardial cell, SMC Svp positive myocardial cells, SN sensory neuron.

Since *tin* is required for *Unc-5* expression in the DV (Fig 1B and 1C). We speculated that *tin* might exert its regulation through the unique DV enhancer we identified within the *Unc-5* genomic locus. Therefore, we examined GFP expression driven by the *Unc-5-GH* enhancer in *tin-ABD*;*tin* ^*346*^ mutant embryos at later stages of embryonic cardiogenesis (Fig 4). GFP expression driven by the enhancer was virtually absent in CBs from *tin-ABD*;*tin* ^*346*^ embryos (from 1.98 ± 0.128 in *wild-type* CBs to 0.173 ± 0.04 in *tin-ABD*;*tin* ^*346*^ CBs, *P*<6 x 10^−14^, Fig 3D). However, GFP expression was still present in PCs (Fig 4B–4B”), where it was slightly reduced (from 3.4 ± 0.2 in *wild-type* to 2.47 ± 0.21 in *tin-ABD;tin* ^*346*^, *P*<6 x 10^−4^, Fig 3E). Importantly, GFP expression from the GH enhancer in a GFP-positive subpopulation of sensory neurons (SNs) where *tin* is not expressed nor required was not affected in *tin-ABD;tin* ^*346*^ mutants (3.86 ± 0.4 in *wild-type* and 3.82 ± 0.29 in *tin-ABD;tin* ^*346*^, Fig 4A” and 4B” [arrowheads], D, E). Furthermore, SMCs, where Tin is not normally expressed, accordingly, still expressed GFP in *tin-ABD;tin* ^*346*^ mutants (S1A–S1A” Fig). These results indicate that *tin* specifically regulates expression through the *Unc-5-GH* enhancer in CBs at the developmental stage when they fuse to form the heart lumen.

### Tin binds the Unc-5 DV enhancer at three conserved Tin-binding elements

**Fig 5:**
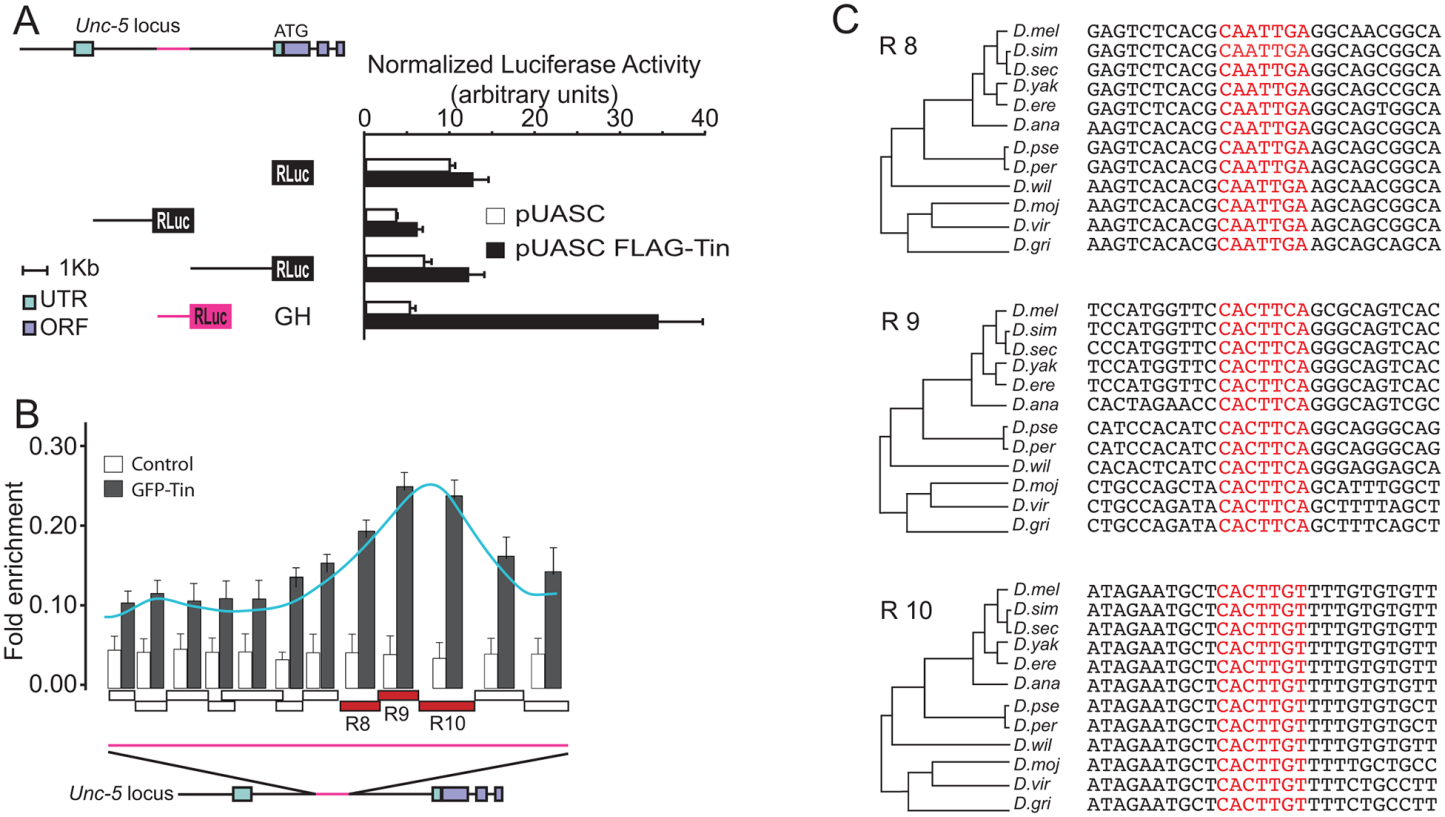
The *Unc-5-GH* enhancer element is directly regulated in vitro by Tin via multiple evolutionary conserved Tin-binding motifs. (A) Tin induces activation of the *Unc-5-GH* enhancer in S2R+ cells. Schematic of a few enhancers within the 5’ region of *Unc-5* used to make luciferase constructs used in the luciferase reporter assays. Luciferase activity was normalized to Firefly activity and the only construct presenting activity corresponds to the *Unc-5-GH* element (magenta). (B) ChIP analysis of the *Unc-5-GH* locus in S2R+ cells transfected with pAct5C-*GFP-tinman*. The precipitated DNA was amplified by real-time qPCR using overlapping primers (boxes on the X axis of the graph) designed to fully cover the identified GH enhancer element (magenta line). Enrichments are presented as percentages of total input and error bars represent the standard deviation. ChIP signal is schematically outlined as a curve peaking at R8, R9, and R10. A schematic of the *Unc-5* locus is also illustrated below the graph. (C) Alignment of these regions against the 12 sequenced *Drosophila* species reveals complete evolutionary conservation of the Tin-binding motifs in R8, R9 and R10 regions of *Unc-5-GH* enhancer (highlighted in red).

To determine if Tin is sufficient to induce transcription through the *Unc-5-GH* enhancer we fused different *Unc-5* enhancers [28] to renilla luciferase ORF and co-transfected them with *tin* in *Drosophila* S2 cells. The only fragment responsive to Tin in this assay was the one containing the GH enhancer (Fig 5A). Therefore, this enhancer is not only under the control of Tin in vivo in the DV, but also responded to it in vitro. Our assay results also suggested that regulation of this enhancer was very likely mediated through a direct interaction with Tin. To test this hypothesis, we performed chromatin immunoprecipitation (ChIP) followed by qPCR using overlapping primers covering the *Unc-5-GH* enhancer. Our ChIP-qPCR results identified a unique enrichment peak near the 3’ end of the *Unc-5-GH* enhancer covering three consecutive amplified regions (referred to as R8, R9, and R10, Fig 5B). Further analysis of the sequence within the peak revealed three potential binding sites on the *Unc-5-GH* enhancer that closely match the described consensus Tin-binding sequence [23, 24, 35]. These motifs are conserved in *Drosophila* species with a divergence time > 10^7^ years, highlighting their functional relevance (Fig 5C). Thus, our ChIP data has identified evolutionary conserved Tin-binding sites within the *Unc-5-GH* enhancer that are likely required for its regulation by Tin.

### Tinman regulates the Unc-5-GH enhancer in vitro through the conserved Tin-binding elements

**Fig 6:**
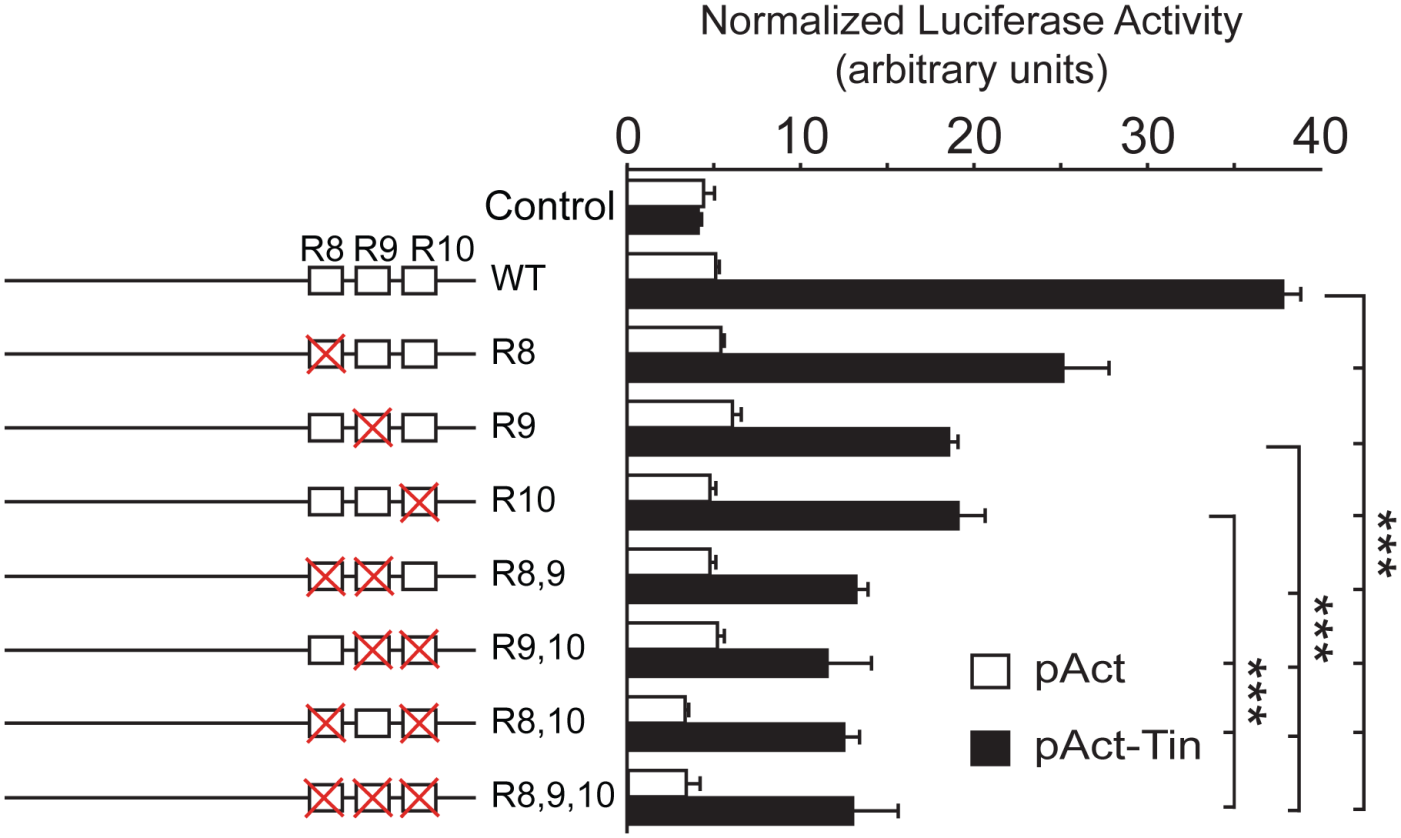
The three Tin-binding motifs in the *Unc-5-GH* element mediate induction of *Unc-5* transcription by Tin in vitro. Mutating the three Tin-binding motifs (singly or in combination) results in reduction of the Tin transcriptional activity as observed in our luciferase assays. Each mutation on a Tin-binding site is represented as a red cross, over the corresponding site (represented as a box) at the left of the graph. Error bars represent the standard deviation and the significance of pairwise comparisons is indicated by *** (p<0.005).

In order to determine the requirement of the identified sites to promote Tin-mediated transcription, we compared the transcriptional activity of the *wild-type Unc-5-GH* enhancer with constructs where each site is changed alone or in combinations (Fig 6). Our in vitro luciferase assay results revealed that mutagenesis of each site lead to reduced transcriptional activity, further confirming that Tin regulates the DV enhancer and its interaction with the conserved binding sites is required to induce *Unc-5* transcription.

### Tinman activity in vivo is mediated through its binding elements on the GH enhancer

**Fig 7:**
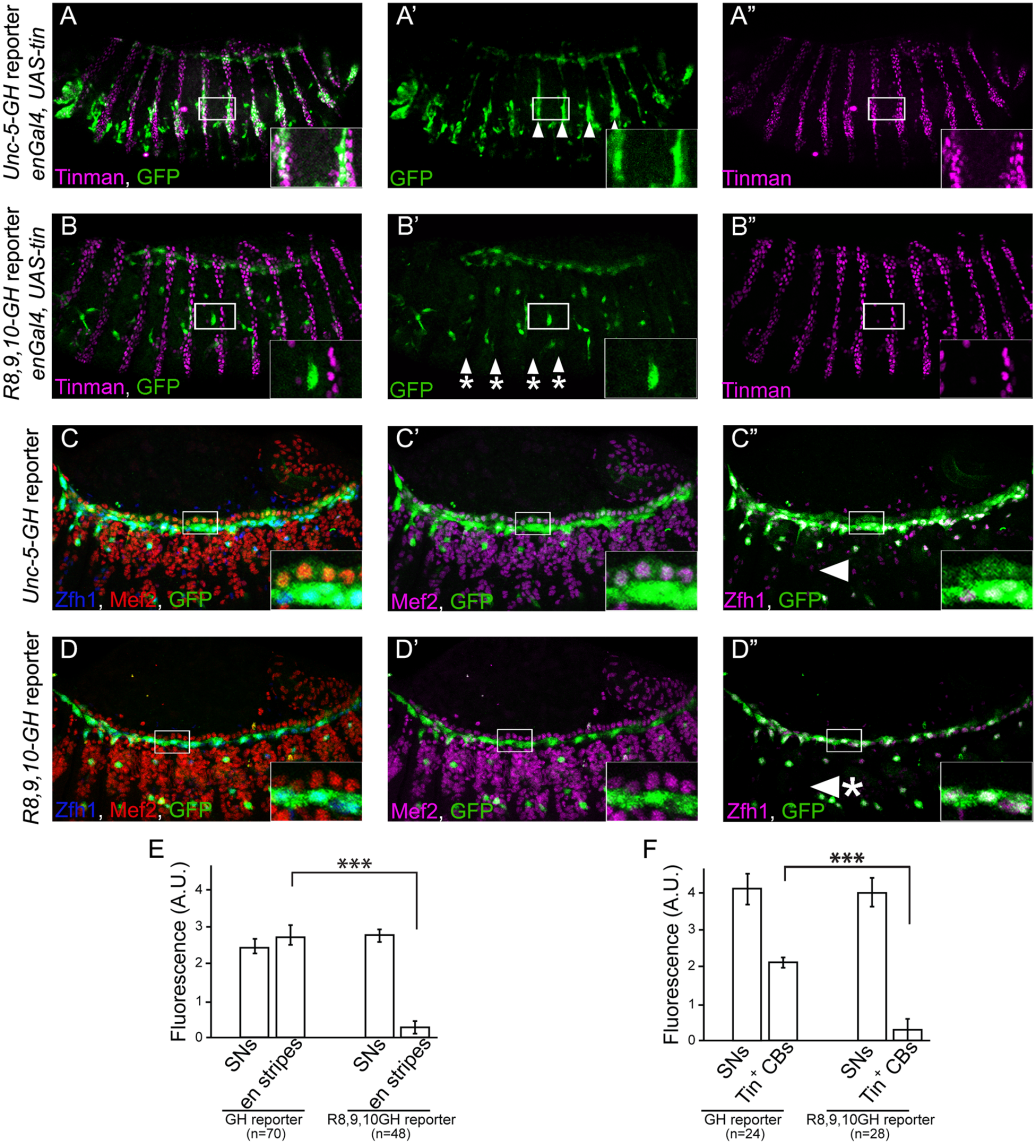
The three Tin-binding motifs in *Unc-5-GH* element mediate induction of *Unc-5* transcription by Tin in vivo. Ectopic expression of *tin* in *engrailed* stripes results in the induction of the *Unc-5-GH* enhancer in a striped pattern (A-A”; arrowheads in A’). Tin expression in ectodermal stripes is labeled with anti-Tin antibody (magenta). Anti-GFP antibody was used to reveal the expression of the reporter (green). As expected, embryos carrying the *R8*,*9*,*10-GH* mutant reporter display little or no GFP induction in the stripes (B-B”; arrowhead-asterisks, and E). (C-C”) The *wild-type Unc-5-GH* enhancer induces expression of the GFP reporter (green) in all CBs and PCs. Mef2 (red, D or magenta, D’) and Zfh1 (blue, D or magenta D”) antibodies are used to reveal CBs or PCs, respectively. The *R8*,*9*,*10-GH* enhancer generates a GFP expression pattern similar to that of the *wild-type Unc-5-GH* enhancer in *tin-ABD; tin* ^*346*^/*tin* ^*346*^ embryos (Fig 4B–4B”) with near complete loss of GFP expression in Tin-positive CBs (D’ and F) and a reduction of expression in PCs (D”). (E and F) Quantification of GFP expression by the mutated *Unc-5* enhancer (*R8*,*9*,*10-GH*) in ectodermal stripes (E) and CBs (F). Genotypes are indicated on the X axis and fluorescence intensities on the Y axis. For all quantifications GFP expression in sensory neurons (SNs) was used as internal control, as the fluorescence in these cells is not affected. In E, fluorescence is significantly reduced (*p*<1.2 x 10^−14^) in *engrailed* stripes of embryos with the mutant reporter compared to those of embryos carrying the *wild-type* reporter [from 2.8 ± 0.26 s.e.m. for the *wild-type Unc-5-GH* reporter to 0.25 ± 0.16 s.e.m. for the *R8*,*9*,*10-GH* reporter (with unchanged SNs’ fluorescence of 2.52 ± 0.192 s.e.m and 2.8 ± 0.177 s.e.m, respectively)]. (F) GFP fluorescence is also significantly reduced (*p*<7 x 10^−18^) in CBs from 1.98 ± 0.127 s.e.m. for the *wild-type Unc-5-GH* reporter to 0.28 ± 0.052 s.e.m. for the *R8*,*9*,*10-GH* mutant reporter. In F, fluorescence in SNs is not affected with unchanged SNs’ fluorescence of 3.75 ± 0.44 s.e.m in *Unc-5-GH* embryos and 3.79 ± 0.36 s.e.m in *R8*,*9*,*10-GH* embryos, respectively. All panels are lateral views of stage 14–15 embryos with dorsal side up and anterior to the left. A magnification of the regions delineated by insets is shown for each panel.

Given that *tin* is sufficient to induce *Unc-5* expression in ectodermal stripes (Fig 2A–2B’), if this regulation is mediated through the DV enhancer, it should also be sufficient to induce ectopic transcription from the GH reporter. As expected, misexpression of the reporter was observed in the *tin*-misexpressing ectoderm (Fig 7A–7A” and 7E). Thus, *tin* is sufficient to induce *Unc-5* expression from its endogenous locus or from a reporter containing the *Unc-5-GH* enhancer in vitro or in vivo. As the *Unc5-GH* enhancer is regulated directly by Tin in vitro (Fig 5) and in CBs in vivo (Fig 4), we reasoned that it might be mediated through the three identified Tin-binding sites in the GH enhancer (Fig 6). To verify this requirement, we misexpressed *tin* in *en* stripes in the presence of a mutant reporter with all three binding sites mutated (*R8*,*9*,*10-GH*). While the *wild-type* reporter is ectopically expressed in *en* stripes our results revealed little or no activity in embryos with the *R8*,*9*,*10-GH* mutant reporter (compare Fig 7A–7A” with Fig 7B–7B” and 7E). Therefore, the ability of Tin to regulate *Unc-5* in vivo, in the ectoderm, is strictly dependent on the conserved Tin-binding sites identified in vitro. Based on these observations we predicted that Tin regulates *Unc-5* through a direct binding to these sites also in CBs. Indeed, GFP expression from the *R8*,*9*,*10-GH* mutant enhancer was also absent from CBs (compare Fig 7C–7C” with Fig 7D–7D” and 7F), indicating that these sites are required by Tin to regulate *Unc-5* in CBs. As our internal control we also determined that reporter expression was not affected in cells that never express nor require Tin such as SNs (Fig 7E and 7F). Together, our results demonstrate that *Unc-5* is regulated by *tin* in cardioblasts through three evolutionarily conserved Tin-binding sites.

## Discussion

Cardiac mesoderm specification is strongly dependent on the combined actions of several transcription factors including the *Doc* family of T-box transcription factors and *tin* [2, 23, 24]. Early mesodermal expression of *Unc-5* is also dependent on the combined actions of *tin* and *Doc* [23] and *Unc-5* cardiogenic mesoderm enhancers are bound by *tin*, *Doc* and *Pnr* [24]. However, at later stages of cardiogenesis their expression pattern segregates; *tin* is restricted to CBs and becomes the major regulator in these cells while *Doc* expression is restricted to SMCs (reviewed in [2]). One of *tin* functions in CBs is to repress *Doc*, and consequently activate only *tin* dependent genes [17]. Our results show that at this developmental stage CB-specific expression of Unc-5 is strictly dependent on *tin* (Fig 1). In *tin-ABD;tin* ^*346*^/ *tin* ^*346*^ mutants all CBs ectopically express *Doc* [17]; however, it does not seem sufficient to promote *Unc-5* expression on them (Fig 1) or through the *Unc-5-GH* heart enhancer (Fig 4). In SMCs where *tin* is not expressed but *Unc-5* is (Fig 3) *Unc-5* is still expressed in *tin* mutants. It is very likely that its expression in these cells is dependent on *Doc* and *svp*. In fact, cardiac mesoderm specification is strongly dependent on the combined action of several transcription factors including the *Doc* family of T-box transcription factors and *tin* [2]. At this stage *Unc-5* expression is dependent on both, *tin* and *Doc* [23]. Thus, *tin* specific regulation of *Unc-5* in CBs when the tubular DV assembles could represent a mark of the original cardiogenic transcriptional code owing to its developmental lineage. It would be interesting to determine if Doc is regulating Unc-5 in SMCs to confirm the segregation the expression pattern of the transcriptional regulators is reflected functionally. Enhancer regulation in CBs, where expression is virtually absent in *tin* mutants, contrasts with that of PCs where is still moderately active (Fig 4) indicating a partial requirement for *tin*. Interestingly, some PCs express *eve*, a known regulator of *Unc-5* in motoneurons [28, 36]. *tin* may work combinatorially with *eve* and other regulators in PCs as shown for *Unc-5* regulation by *eve* in motoneurons [37].

It has been recently shown that Unc-5 receptor’s role during heart morphogenesis is to preserve the luminal space between opposing CB membranes during heart tube lumen formation [7, 8]. Accordingly, *Unc-5* and the *Unc-5*-*GH* reporter are expressed during tubulogenesis (Fig 3A–3E”) and its expression in CBs is strictly dependent on *tin* (Fig 1). Thus, there is a perfect match between *Unc-5* expression in CBs and *tin* regulation. Furthermore, the elimination of the Tin-binding sites in the DV enhancer renders it unresponsive to Tin in vitro (Fig 6) and in vivo (Fig 7). Therefore, our results strongly suggest that *Unc-5* is specifically regulated by *tinman*, through a direct interaction with three evolutionary conserved sites within its regulatory region at later stages of DV tubulogenesis.

Given the high degree of conservation on the molecular pathways controlling heart morphogenesis in vertebrates [38, 39] the NK2, family of transcription factors is a very likely candidate to drive this process, in part, through a direct regulation of guidance receptors.

## Materials and Methods

### Genetics

The following stocks were used: *Tin-ABD*; *tin* ^*346*^ */TM3*, *eve-lacZ*, svp-*lacZ* [17], *en-Gal4*, *tup* ^*isl-1*^ */Cyo*, *pnr* ^*1*^ */TM3*, pan^3^/Dp(2;4)ey^D^, Alp^eyD^: ^eyD^, *Unc-5 GH-GFP*, *Unc-5* GH-Gal4 (described below), *TinC-Gal4* [40], and *UAS-tau-Myc*.

### Generation of constructs

*Unc-5* locus dissection was carried out by PCR-amplifications using genomic DNA as template to amplify overlapping fragments of random sizes. The PCR products were cloned using TOPO TA Cloning (Invitrogen), sequenced and recombined into destination vectors: *pGateway-nlsVenus-attB* and/or *pGateway-Gal4* and integrated into the attP2 site [41]. The *pGateway-Rluc* vector was used for luciferase assays. PCR amplified *tin* was cloned into pActC-GFP or pAct5C-FLAG plasmids to generate GFP-Tin and FLAG-Tin used in ChIP or luciferase assays, respectively. For site directed mutagenesis of Tin binding sites the most conserved nucleotides within the CACTTGA consensus motif, the “CA” dinucleotide and the first “T” [24], were mutated to “GT” and “A“, respectively. The following primers were used for mutagenesis: CACGGTATAGAGGCAACGG and CCGTTGCCTCTATACCGTG for R8, GTTCGTCTACAGGGCAGTCAC and GTGACTGCCCTGTAGACGAAC for R9, and TGCTGTCTAGTTTTGTGTGTTCTG and CAGAACACACAAAACTAGACAGCA for R10.

### Immunohistochemistry and mRNA in situ hybridization

Embryo collection, immunohistochemistry and in situ hybridization were performed as previously described [28]. Reporter gene (GFP) expression in DV was quantified at embryonic stages 14–16 in different genetic backgrounds. The following antibodies were used: Mef2 (1:2000), Zfh1 (1:1500), Odd (1:1000) [33], Tin (1:1000) [27], chicken anti-GFP (1:1500) (ab13970; Abcam). Eve (1:50), c-Myc 9E10 (1:50), β-gal (1:50) were purchased from DHSB. Secondary antibodies: Alexa 555, Alexa 488-conjugated (Invitrogen) and Cy5-conjugated (Jackson ImmunoResearch Laboratories). *Unc-5* in situ hybridization was performed with digoxigenin-labelled probes as previously described [21]. HRP-conjugated anti-digoxigenin (Roche) followed by incubation with Cy3-labelled tyramide (PerkinElmer), as substrate, was used for detection of the hybridized probes.

Stacks of images were obtained using Zeiss Confocal LSM700 Microscope and 20X or 40X oil-immersion. ImageJ was used for quantification of fluorescence within regions of interest (ROI). For GFP fluorescence quantification, all controls and samples were fixed together using the same procedure, stained with GFP antibodies and imaged using the same configurations. Samples and controls were mounted in the same slide for imaging. Image analyses were done using ImageJ software. Background correction was performed individually for each embryo and the intensity for GFP ROIs was divided by the intensity of control areas and finally averaged for each genotypic group.

### Statistical analysis

Statistical significance of alterations in luciferase activity levels for reporters with different mutations or fluorescence intensity in different samples were calculated using one-tailed t-test for pair-wise comparisons and histograms were generated using Microsoft Excel 2013.

### Chromatin immunoprecipitation

ChIP was performed and analyzed essentially as described previously [42]. In summary, extracts from S2R+ cells transfected with either *pAct5C-GFP-Tin* or *pAct5C* (as mock control) were fixed in 1% formaldehyde for 10 minutes at room temperature and then lysed. Following shearing the chromatin by sonication, lysates were incubated with rabbit anti-GFP (ab290; Abcam) for 2 hours at 4°C followed by incubation with protein A-sepharose (P9424; Sigma) for an additional 2 hours. Beads were then washed and the immunoprecipitated material were eluted at 70°C overnight. Phenole-chloroform DNA extraction was performed the next day to purify the precipitated DNA. The immunoprecipitated DNA was subsequently quantified by real-time qPCR.

### Luciferase reporter Assays

S2R+ cells were used for luciferase assays. Approximately 10^5^ cells were transfected (using FuGENE® HD Transfection Reagent, E2311) with the transcription factor plasmid, Rluc construct, and PolIII-Fluc (as internal control). Cells were analyzed for luciferase activity 36 hours post transfection using the Dual-Glo Luciferase Kit (Promega) according to manufacturer’s instructions. Samples were assessed in triplicate.

## Supporting Information

S1 Fig Reporter expression in SMCs. In *tin-ABD; tin* ^*346*^/*tin* ^*346*^ mutant background a few CBs maintain reporter gene expression (inset in A’, arrowhead). LacZ co-staining, in the presence of Svp-LacZ reporter, (blue in A or magenta in A”) indicates that these are Tin-negative, Svp-positive CBs (SMCs).
(TIF)
