# Supplementary figures and images for: The Unc-5 Receptor Is Directly Regulated by Tinman in the Developing Drosophila Dorsal Vessel
Source: PLoS One. 2015 Sep 10;10(9):e0137688. doi: 10.1371/journal.pone.0137688 (PMC4565662; doi:10.1371/journal.pone.0137688)

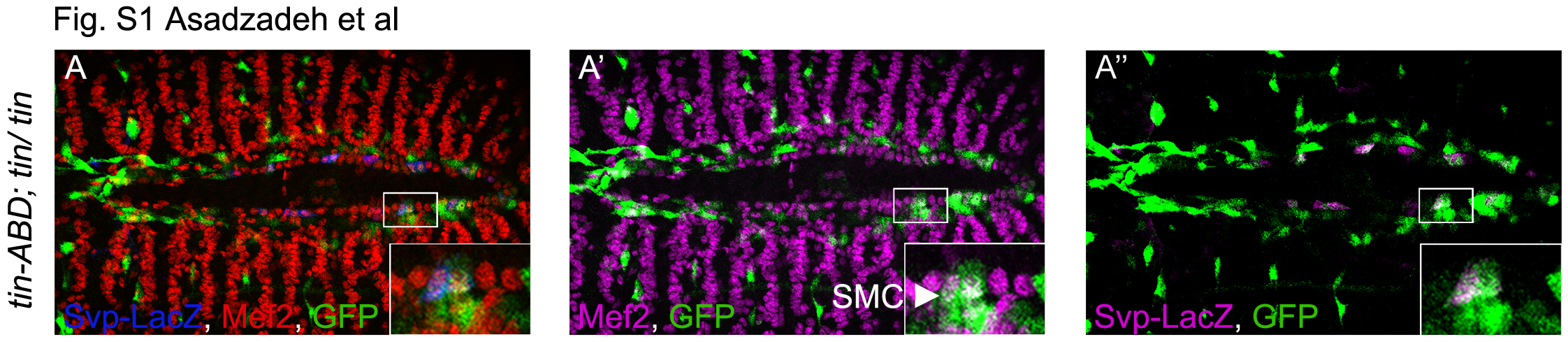

Supplement: S1 Fig — In tin-ABD; tin 346/tin 346 mutant background a few CBs maintain reporter gene expression (inset in A’, arrowhead). LacZ co-staining, in the presence of Svp-LacZ reporter, (blue in A or magenta in A”) indicates that these are Tin-negative, Svp-positive CBs (SMCs). (TIF) [file pone.0137688.s001.tif]
